# Facultative Symbiont Infections Affect Aphid Reproduction

**DOI:** 10.1371/journal.pone.0021831

**Published:** 2011-07-27

**Authors:** Jean-Christophe Simon, Sébastien Boutin, Tsutomu Tsuchida, Ryuichi Koga, Jean-François Le Gallic, Adrien Frantz, Yannick Outreman, Takema Fukatsu

**Affiliations:** 1 INRA, UMR 1099 INRA-Agrocampus Ouest-Université de Rennes 1 “Biologie des Organismes et des Populations appliquée à la Protection des Plantes” (BiO3P), Le Rheu, France; 2 Bioproduction Research Institute, National Institute of Advanced Industrial Science and Technology (AIST), Tsukuba, Japan; 3 Toyama Support Center for Young Principal Investigators in Advanced Life Sciences, University of Toyama, Toyama, Japan; 4 UPMC Université Paris 06, UMR 7625, Ecologie & Evolution, Paris, France; University of Poitiers, France

## Abstract

Some bacterial symbionts alter their hosts reproduction through various mechanisms that enhance their transmission in the host population. In addition to its obligatory symbiont *Buchnera aphidicola*, the pea aphid *Acyrthosiphon pisum* harbors several facultative symbionts influencing several aspects of host ecology. Aphids reproduce by cyclical parthenogenesis whereby clonal and sexual reproduction alternate within the annual life cycle. Many species, including the pea aphid, also show variation in their reproductive mode at the population level, with some lineages reproducing by cyclical parthenogenesis and others by permanent parthenogenesis. While the role of facultative symbionts has been well studied during the parthenogenetic phase of their aphid hosts, very little is known on their possible influence during the sexual phase. Here we investigated whether facultative symbionts modulate the capacity to produce sexual forms in various genetic backgrounds of the pea aphid with controlled symbiont composition and also in different aphid genotypes from natural populations with previously characterized infection status and reproductive mode. We found that most facultative symbionts exhibited detrimental effects on their hosts fitness under sex-inducing conditions in comparison with the reference lines. We also showed that the loss of sexual phase in permanently parthenogenetic lineages of *A. pisum* was not explained by facultative symbionts. Finally, we demonstrated that *Spiroplasma* infection annihilated the production of males in the host progeny by inducing a male-killing phenotype, an unexpected result for organisms such as aphids that reproduce primarily through clonal reproduction.

## Introduction

Many organisms engage in symbiotic relationships with a wide range of microbes. These sustained and intimate associations provide conditions for the evolution of complex phenotypes on which natural selection may operate and favor one or both partners of the symbiosis. Such symbionts frequently modify the ecology and physiology of their hosts to enhance their own transmission. This involves a variety of mechanisms that either increase host fitness or interfere with host reproductive biology, including sex ratio distortion [1], [2].

Aphids represent the best-studied case of such symbiotic associations. First, these insects established an obligate symbiosis with the endocellular bacterium *Buchnera aphidicola* 150–200 million years ago [3]. *Buchnera* provides the host with essential amino acids lacking in the host diet and may also be involved in thermal adaptation in certain circumstances [4]. In this case of obligate symbiosis, inheritance of bacterial symbionts is strictly vertical and both partners are mutualistically dependent on each other. In addition to *Buchnera*, aphids may also harbor one or several facultative symbionts belonging to distinct bacterial lineages. These facultative symbionts have been mostly studied in the pea aphid *Acyrthosiphon pisum*, whereas such microorganisms have been screened in many more aphid species [5]–[7]. In the pea aphid, seven facultative symbionts have been identified so far: *Hamiltonella defensa*, *Regiella insecticola*, *Serratia symbiotica*, *Rickettsiella* sp. and so-called PAXS representing the *Gammaproteobacteria*; *Rickettsia* sp. of the *Alphaproteobacteria*; and *Spiroplasma* sp. of the *Mollicutes* [8]–[14]. Other facultative symbionts including *Arsenophonus* and *Wolbachia* are found in some aphid species but not in the pea aphid [14], [15]. The community of facultative symbionts within a host can be manipulated to create experimental lines with the same aphid genotype but different symbionts, by selective elimination with antibiotics, and transfection from infected to uninfected aphids [8], [13], [16], [17]. Such experimental approaches have been essential for investigating the effects of facultative symbionts on several aspects of their hosts' ecology. In the pea aphid, such effects include host plant utilization [17], body color change [13], heat tolerance [18] and protection against natural enemies [12], [19]–[21].

Aphids reproduce by cyclical parthenogenesis wherein clonal and sexual reproduction alternate within the annual life cycle. Many aphid species, including *A. pisum*, also encompass some lineages that have lost the sexual phase and reproduce by permanent parthenogenesis [22]. Some of the facultative symbionts reported in aphids are known to alter reproduction in other arthropod hosts, with *Rickettsia*, *Spiroplasma* and *Wolbachia* inducing various reproductive phenotypes such as male-killing, feminization and parthenogenesis in a wide range of arthropods [1], [2]. Thus far, biological and ecological roles of facultative symbionts have been well studied during the parthenogenetic phase of their aphid hosts, but very little is known on their possible influence during the sexual phase [23]–[25]. Here we investigated whether facultative symbionts modulate the capacity to produce sexual forms under various genetic backgrounds of the pea aphid with controlled symbiont compositions.

## Materials and Methods

### Selection of pea aphid genotypes

Ten pea aphid genotypes of *A. pisum* collected mainly in western and central France were selected for generating manipulated lines with known symbiont compositions (Table 1). These genotypes were subjected to previous studies on reproductive mode variation and ecological specialization in the pea aphid, wherein their genotyping and symbiont typing had been completed [26]–[29]. Four genotypes originating from pea or clover were used as sexual genotype representatives: i.e. they produced sexual females and males under sex-inducing conditions (see below). Five other genotypes originating from alfalfa or clover were chosen as asexual genotype representatives: i.e. they produced no sexual females under the sex inducing conditions while male production rates may be variable [27].

**Table 1:** Biological characteristics of the pea aphid genotypes used for the construction of lines with manipulated symbiotic composition.

Clone name	Color	Plant origin	Location	Collection date	Original infection status	Reproductive mode
T3-8V1	Green	Clover	Domagné, France	June 2003	*Regiella* (donor)	Permanent parthenogenesis
10TV	Pink	Clover	Domagné, France	June 2002	*Regiella*	Permanent parthenogenesis
4TV	Pink	Clover	Domagné, France	June 2002	*Regiella*	Permanent parthenogenesis
YR2	Pink	Clover	York, UK	Dec. 2002	*Regiella*	Cyclical parthenogenesis
L1-22	Green	Alfalfa	Domagné, France	June 2002	*Hamiltonella*	Permanent parthenogenesis
L12	Green	Alfalfa	Domagné, France	June 2002	*Spiroplasma* (donor) + *Hamiltonella*	Permanent parthenogenesis
L100	Green	Alfalfa	Lusignan, France	March 1999	*Hamiltonella* (donor)	Permanent parthenogenesis
P33	Green	Pea	Le Rheu, France	June 2002	*Rickettsia*	Cyclical parthenogenesis
P123	Green	Pea	Mauzé-le-Mignon, France	April 1999	*Rickettsia* (donor)	Cyclical parthenogenesis
P136	Green	Pea	Mauzé-le-Mignon, France	April 1999	*Serratia* (donor) + *Rickettsiella*	Cyclical parthenogenesis

Donors used for transfection experiments of facultative symbionts are shown in parentheses.

### Symbiont elimination and transfection

Facultative symbionts harbored by the tested pea aphid genotypes were selectively eliminated by antibiotic treatments. Selective elimination of *Hamiltonella* was conducted by treatment of a mixture of ampicillin, cefotaxime and gentamycin with minor modification of published procedure [30]: injection of each at a dose of 250 µg/ml was conducted instead of treatment by feeding artificial diet containing the antibiotics. Selective elimination of *Serratia*, *Regiella* and *Rickettsia* was performed by ampicillin injection as described in [17], [31], [32]. We repeatedly attempted different cocktails and doses of the antibiotics, but failed to eliminate *Spiroplasma* in line L12 without affecting *Buchnera* infection. Genotype L12 was thus not included in the following experiments. Transfection of each of the five facultative symbionts (*Hamiltonella*, *Regiella*, *Rickettsia*, *Serratia* and *Spiroplasma*) was conducted by hemolymph injection using a glass microcapillary as described in [31]. The donors for the facultative symbionts are listed in Table 1. It should be noted that P136, which was used as *Serratia* donor, also harbored *Rickettsiella*, a newly reported bacterium that was discovered incidentally by us after the establishment of manipulated lines tested here [13]. Hence, strains injected with hemolymph of P136 received both *Serratia* and *Rickettsiella* symbionts. All the recipient aphid strains were treated with the antibiotics to eliminate pre-existing facultative symbionts, and successful elimination was checked by diagnostic PCR. Newborn nymphs deposited by the injected mothers were collected 13 days after injection and reared individually, from which isofemale lines (i.e. parthenogenetic/clonal lines with the same genetic background but different in their facultative symbiont infection) were established. Infection statuses of 16 newborn nymphs in each isofemale line were checked by diagnostic PCR for three successive generations. All individuals exhibited the same symbiotic profile. The stability of infection by facultative symbionts was checked periodically over 10 generations. A total of 9 (genotypes)×6 (infection status) = 54 isofemale lines were obtained and used for subsequent sex induction experiments that were performed six to eight months after the treatments (equivalent to about 18 to 24 generations). Genotype identity and infection status of each line were checked prior to biological experiments (for technical details see [25], [29]).

### Sex induction experiments

In the first experiment, we tested the 54 isofemale lines in “standard” controlled conditions that trigger sexual form production in the pea aphid [25]. Here, we intended to assess the effect of each of the facultative symbionts on sex-related life-history traits of their hosts. For that purpose, the manipulated lines obtained from the sexual and asexual genotypes were exposed to a short-day regime (12 h light and 12 h dark) at 18°C throughout the experiment. These conditions generally induce sexual females in sexual genotypes after two generations, but are sometimes insufficient to induce males in large numbers [33]. The experiment was repeated 12 times for each of the lines. For each of the replicates, a third instar nymph (generation G0) was isolated from the stock culture, placed on a broad bean plant and exposed to 12 h light and 12 h dark cycles at 18°C. The first-born nymph (generation G1) was isolated and reared under the same conditions, which developed into a parthenogenetic female and produced offspring (generation G2).

In the second experiment, we used a modified protocol that enhances male production in the pea aphid. Here we intended to explore in more detail the effect of *Rickettsia* and *Spiroplasma*, two microbes that had been known to induce male-killing phenotypes in a variety of arthropods, on the four sexual genotypes. For each of the genotypes, male production of *Rickettsia-*infected and *Spiroplasma*-infected lines was compared with that of reference lines infected with *Buchnera* only. The experiment 2 was repeated 12 times for each of the lines. In contrast to the experiment 1, G0 individuals in the experiment 2 were first exposed to a short day regime (12 h light and 12 h dark) at 18°C for seven days, while subsequent generations (G1 and G2) were placed under a long-day regime (16 h light and 8 h dark) at 18°C for the rest of the experiment. This treatment enhanced male production and reduced sexual female production [33].

In both experiments, the morph of each of the G2 individuals was determined as parthenogenetic female, sexual female or male based on morphological criteria [25]. Four life history traits were also measured for each of the G1 females: age at first reproduction (i.e. time from birth to onset of reproduction), total fecundity (number of offspring), reproductive lifespan (i.e. time from first to last offspring production) and longevity (i.e. time from birth to death).

### Statistical analyses

In both experiments, each response was analyzed against the infection status of the aphid, the genotype of the aphid, and the interaction between these two independent variables using generalized linear models (GLMs). In these statistical models, both the infection status and the genotype of the aphids were considered as fixed factors (i.e., class independent variables). The link function and the error distribution used in the GLMs depended on the analysed response (i.e., a logit link function and a quasibinomial error distribution were used for binomial response; a log link function and a quasipoisson error distribution were specified for counting data; an inverse function and a gamma error distribution were used for duration data). Both quasipoisson and quasibinomial error distributions were used to deal with overdispersed data. Models were simplified to the minimal adequate model by stepwise removing of non-significant elements. In case that the infection status exhibited significant effects, the level of response for aphids harbouring one of the five facultative symbionts was compared with that for aphids without the facultative symbiont (i.e., reference *Buchnera* line). For this purpose, contrast tests were performed by using the function ‘*esticon*’ in the ‘*doBy*’ add-on package (Author: Søren Højsgaard). The same statistics were computed on the data previously obtained for naturally infected lines from pea aphid field populations (see results). All the analyses were done using the program package R [34].

## Results

In the experiments, strong aphid genotype×symbiont interactions were identified for all the life history traits, i.e. age at first reproduction, total fecundity, reproductive lifespan and longevity, and variables related to morph production, i.e. proportions of asexual females and males in the progeny (Table S3). In other words, the effects of the facultative symbiont infections on fitness components of their hosts varied according to the tested pea aphid genotypes. In addition, the aphid genotype and the infection status individually had a significant influence on each of the variables. In the following sections, we detailed the results of the experiments 1 and 2 for three representative variables: longevity, total fecundity and male production in the progeny (Table 2). Means and standard errors of the whole set of variables are given in Table S1 and Table S2 for experiments 1 and 2, respectively.

**Table 2:** Generalised linear models showing the effects of facultative symbionts infection status and the genotypes of *Acyrthosiphon pisum* on their reproductive life history traits.

		Model elements								
		Aphid infection status (1)			Aphid genotype (2)			Interaction (1)×(2)		
	Dependent variables (distribution family)	*d.f.*	*F*	*P*-value	*d.f.*	*F*	*P*-value	*d.f.*	*F*	*P*-value
Experiment 1										
	*(a) Asexual genotypes*									
	Longevity (Gamma)	5	125.41	<0.001	4	33.45	<0.001	20	10.72	<0.001
	Total fecundity (Quasipoisson)	5	76.77	<0.001	4	83.11	<0.001	20	7.55	<0.001
	*(b) Sexual genotypes*									
	Longevity (Gamma)	5	131.69	<0.001	3	4.87	<0.005	15	12.14	<0.001
	Total fecundity (Quasipoisson)	5	76.31	<0.001	3	34.27	<0.001	15	5.97	<0.001
	Proportion of males (Quasibinomial)	5	59.35	<0.001	3	264.60	<0.001	15	2.67	<0.001
Experiment 2										
	Total fecundity (Quasipoisson)	2	110.38	<0.001	3	3.02	<0.05	6	12.33	<0.001
	Proportion of males (Quasibinomial)	2	123.93	<0.001	3	102.27	<0.001	6	4.19	<0.001

### First experiment

**Figure 1:**
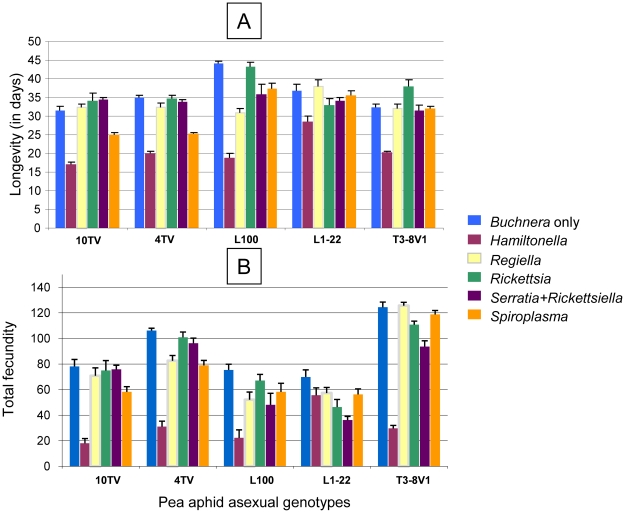
Influence of symbiont component on history traits of artificially infected lines of five asexual genotypes of the pea aphid exposed to standard conditions of sex induction (experiment 1). A) longevity and B) fecundity.

**Figure 2:**
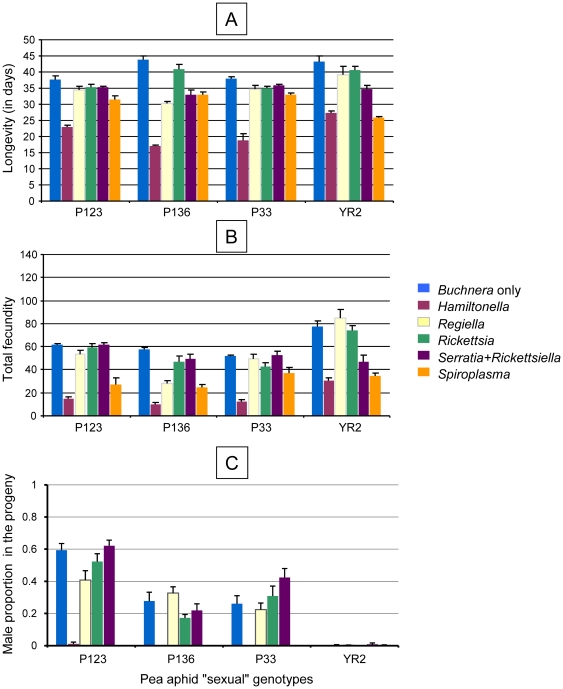
Influence of symbiont component on history traits of artificially infected lines of four “sexual” genotypes of the pea aphid exposed to standard conditions of sex induction (experiment 1). A) longevity, B) fecundity and C) sex ratio (given as the proportion of females in the sexual fraction of the progeny).

The influence of each of the facultative symbionts was assessed by the contrast method, using the *Buchnera* only treatment as reference (Table 3). In many instances, the facultative symbionts exhibited a significantly negative impact on their host fitness. *Rickettsia* exhibited the smallest effect on the host: no influence on longevity and total fecundity of the asexual genotypes and a moderately negative effect on longevity of the sexual genotypes (Table 3). By contrast, *Hamiltonella* exhibited the greatest detrimental effect on various fitness components in both the asexual and sexual genotypes, with a 60% fecundity reduction on average (Figures 1 and 2). *Spiroplasma* also exhibited a negative impact on the host fitness, which was stronger in the sexual genotypes than in the asexual genotypes, reducing total fecundity of the latter by 47% on average.

**Table 3:** Singular effects of facultative symbionts on different life history traits of *Acyrthosiphon pisum*.

		Facultative symbionts				
	Dependent variables	*Hamiltonella*	*Regiella*	*Rickettsia*	*Serratia* + *Rickettsiella*	*Spiroplasma*
Experiment 1						
	*(a) Asexual genotypes*					
	Longevity	<0.001 (−)	<0.05 (−)	0.549 ( = )	0.067 ( = )	<0.001 (−)
	Total fecundity	<0.001 (−)	<0.05 (−)	0.079 ( = )	<0.005 (−)	<0.05 (−)
	*(b) Sexual genotypes*					
	Longevity	<0.001 (−)	<0.001 (−)	<0.05 (−)	<0.001 (−)	<0.001 (−)
	Total fecundity	<0.001 (−)	<0.05 (−)	0.118 ( = )	<0.05 (−)	<0.001 (−)
	Proportion of males	<0.001 (−)	0.128 ( = )	0.461 ( = )	0.056 ( = )	<0.001 (−)
Experiment 2						
	Total fecundity	-	-	0.206 ( = )	-	<0.001 (−)
	Proportion of males	-	-	0.231 ( = )	-	<0.001 (−)

Significance of contrast tests comparing the dependent variable between the reference treatment (no facultative symbiont) with the corresponding symbiont. In brackets, the sign indicates in which manner the symbiont affects the dependent variable (+: symbiont induces an increase in the dependent variable; −: symbiont induces a decrease in the dependent variable;  = : symbiont has no effect on the dependent variable).

Under the conditions where sexual forms are normally induced in the pea aphid, the five tested asexual genotypes produced no sexual females and only a few (<5%) or no males (Table S1) as expected for this type of aphid population [27]. Only L100 showed a relatively high male production, with up to 14% of the progeny with this gender (Table S1). Interestingly, no male production was observed in the progeny of L100 insects infected with *Spiroplasma*. When the four sexual genotypes under the reference treatment (*Buchnera* only) were compared with each other, strong differences were found in their male production: P123 produced up to 62% males in the progeny; P136 and P33 produced some 25% males; and YR2 produced virtually no males.

The specific effects of each of the five facultative symbionts on sexual form production are shown in Table 3. *Regiella*, *Rickettsia* and *Serratia + Rickettsiella* exhibited no significant influence on the proportion of males in the progeny of their host (Figure 2). By contrast, both *Hamiltonella* and *Spiroplasma* exhibited a significant negative effect on male production. To assess whether the effects of the facultative symbionts on reproductive morph production were due to sex ratio manipulation or lower survival, we analyzed the kinetics of female and male productions in the reference vs. infected lines for each of the genotypes. Results showed that male production peaked on average after 8 days of reproduction. *Hamiltonella*-infected lines exhibited a considerably shortened reproductive lifespan (8 days on average; more than a week for only 4 of 9 lines) in comparison with the reference lines, suggesting an indirect influence on male production via shortened reproductive lifespan of their host. By contrast, *Spiroplasma*-infected lines did not exhibit such a drastic life shortening, and thus early mortality cannot account for the complete lack of male production (Table 4).

**Table 4:** Reproductive lifespan (mean and standard error) of pea aphid genotypes in experiments 1 and 2 according to symbiotic composition.

	Reproductive lifespan			
	Experiment 1		Experiment 2	
Infection status	mean	standard error	mean	standard error
*Buchnera* only	20.56	2.93	20.29	2.25
*Hamiltonella*	8.39	3.78	NA	NA
*Regiella*	16.07	4.16	NA	NA
*Rickettsia*	19.81	3.76	17.79	3.10
*Serratia *+* Rickettsiella*	17.51	1.85	NA	NA
*Spiroplasma*	15.29	3.66	11.90	3.08

### Second experiment

**Figure 3:**
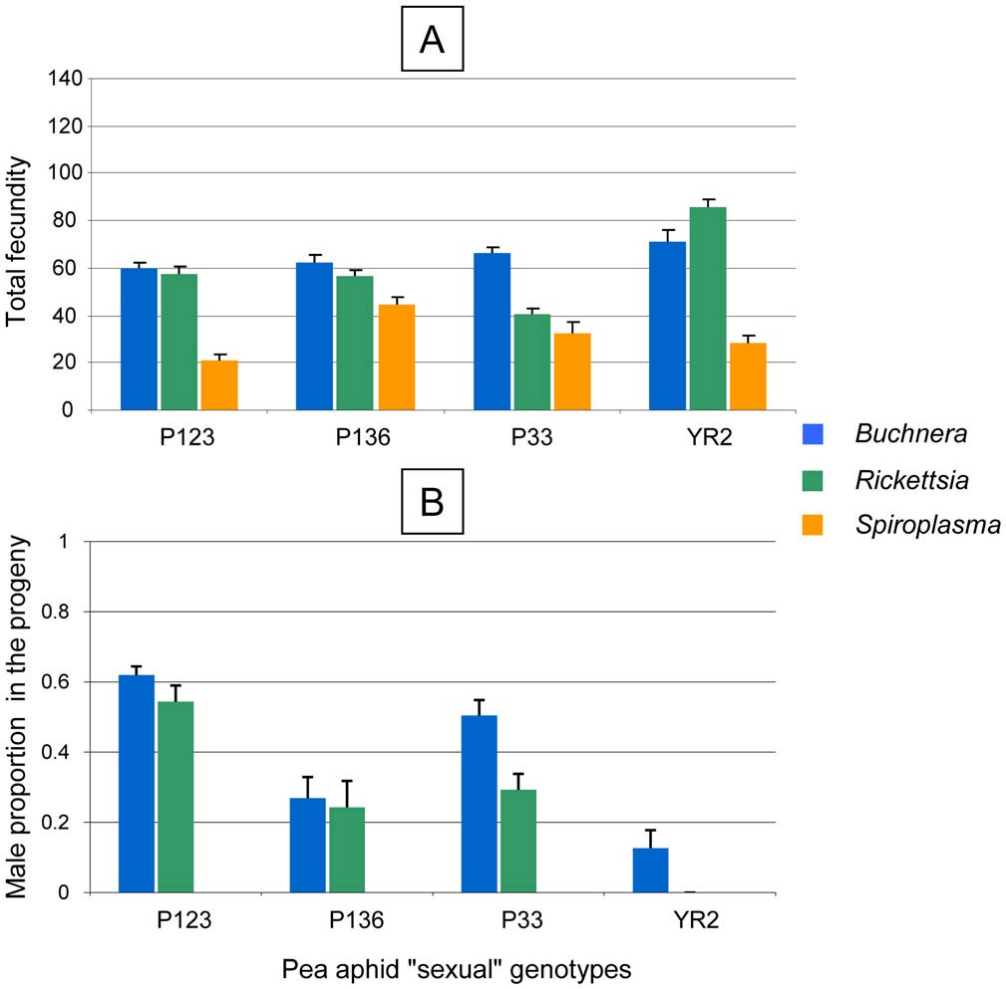
Influence of symbiont component on history traits of artificially infected lines of four “sexual” genotypes of the pea aphid exposed to conditions increasing male production (experiment 2). A) fecundity and B) sex ratio (given as the proportion of females in the sexual fraction of the progeny).

In the experiment 2, male production in the reference lines ranged from 62% in P123 (vs. 59% in the experiment 1) to 13% in YR2 (vs. 0% in the experiment 1). Overall, male production was increased by 10% in the experiment 2 in comparison with the experiment 1 (Table S2). *Rickettsia* had no influence on total fecundity, while *Spiroplasma* exhibited a strong negative impact on it, confirming the results of the experiment 1. Male reduction in *Rickettsia*-infected lines ranged from 100% in YR2 to no reduction in P123 in comparison with their respective reference lines (Figure 3). However, contrast tests did not detect significant effects of *Rickettsia* on male proportions (Table 3). Strikingly, all four sexual genotypes produced no males when infected with *Spiroplasma*, confirming results of experiment 1. In experiment 1, male production in the *Spiroplasma*-infected lines was not affected by early mortality, because of sufficient time to reach the peak of male production (Table 4).

### Male-biased mortality in *Spiroplasma*-infected lines

In both experiments, *Spiroplasma*-infected lines showed a higher mortality of their progeny at early nymphal stages in comparison with their reference lines (S. Boutin, J.F. Le Gallic and J.C. Simon, personal observations). To assess whether this nymphal mortality was sex-specific or not, we collected the dead young nymphs from the four sexual genotypes, and determined their gender using microsatellite markers. Female aphids carry two copies of X chromosomes (XX type) while male aphids have only one (X0 type). Hence, X-linked heterozygous markers should exhibit two distinct alleles in females and one of the alternative alleles in males. On account of the previously characterized X-linked markers [35], we selected a set of heterozygous microsatellites in the four sexual genotypes. We then genotyped the dead nymphs individually at each of the heterozygous loci [36], and determined their gender based on the presence of one (male) or two (female) alleles. Of 94 dead nymphs examined, 92 individuals (98%) were diagnosed as male (Table 5), favouring the hypothesis that *Spiroplasma* induces a male-killing phenotype in *A. pisum*.

**Table 5:** Gender determination of dead larvae in *Spiroplasma*-infected lines for four “sexual” genotypes of the pea aphid.

*Spiroplasma*-infected lines	Dead males	Dead females	Total
P33	28	0	28
P123	30	0	30
P136	16	0	16
YR2	18	2	20
**Total**	**92**	**2**	**94**

### Analyses of male production in naturally-infected lines

In the light of the above results, we evaluated the possible influence of facultative symbionts on male production by re-analyzing the data published in previous studies on reproductive mode and symbiotic composition of 210 pea aphid lines [27]–[29]. This analysis revealed a significant effect (p = 0.004) of *Spiroplasma* on male production: only 50% of the lines infected with *Spiroplasma* were able to produce males, while this proportion was over 80% for lines infected with *Buchnera* only (Table 6). Furthermore, the male-producing lines infected with *Spiroplasma* showed a two-fold reduction in male production in comparison with the lines infected with *Buchnera* only (Table 6). Meanwhile, the other facultative symbionts (including *Rickettsia*) did not affect male production in the pea aphid lines, when compared with the aphid lines infected with *Buchnera* only (p>0.05).

**Table 6:** Proportion of male-producing lines and male production in relation with symbiotic composition among natural populations of the pea aphid (data compiled from [25]).

Infection status	# of pea aphid lines	% of male-producing lines	% of male production	
			mean	SD
*Buchnera* only	39	82.05%	22.09%	0.04
*Hamiltonella*	39	66.67%	14.75%	0.02
*Regiella*	28	71.43%	13.93%	0.02
*Rickettsia*	21	80.95%	34.15%	0.08
*Serratia*	45	84.44%	35.92%	0.05
*Spiroplasma*	38	50.00%	7.92%	0.01

N = 210, SD = standard deviation.

## Discussion

Here we simultaneously analyzed the influence of several symbionts on their hosts' reproduction, and correlated the results on genetically identical aphid lines that differ in their symbiont contents with the results from natural aphid populations. While recent works on facultative symbionts of the pea aphid have shown their important and diverse roles on the ecology and physiology of their host, the possibility that they may manipulate directly their host reproductive biology has been largely neglected.

### *Spiroplasma* induces a male-killing phenotype in pea aphid populations

Injection of a *Spiroplasma* strain into several genetic backgrounds of the pea aphid consistently inhibited male production in two independent experiments. We demonstrated that the absence of males in the progeny of *Spiroplasma*-infected females is not likely due to shortened lifespan that would truncate the reproductive sequence. Instead, *Spiroplasma* infection induced male-specific mortality at early nymphal stages as revealed by sex identification using microsatellite markers. Furthermore, the possibility of *Spiroplasma*-induced male-killing was also suggested by a large quantity of data previously obtained from natural populations of the pea aphid: the proportion of male-producing genotypes was reduced on average by half in *Spiroplasma*-infected clones. All these results consistently indicate a male-killing phenotype induced by *Spiroplasma* infection, which has never been reported from aphids. Male-killing bacteria have been identified from such groups like *Spiroplasma* in the *Mollicutes*, unnamed species in the *Flavobacteria*, *Rickettsia* and *Wolbachia* in the *Alphaproteobacteria*, and *Arsenophonus nasoniae* in the *Gammaproteobacteria* [1]. Interestingly, male-killing phenotypes caused by *Spiroplasma* have been frequently found among ladybirds, a major group of aphid predators, and the *Spiroplasma* strains infecting the pea aphid and ladybirds are phylogenetically very close to each other [37]. Whether these prey-predator interactions may favour horizontal transfers of *Spiroplasma* as shown for other trophic systems [38], [39] awaits validation.

Distinct from the other reported cases of symbiont-induced male-killing, the *Spiroplasma*-induced male-killing of the pea aphid is expressed during a small part of its life cycle, because most of pea aphid generations are asexual. If male-killing does maintain *Spiroplasma* infection in natural populations of *A. pisum*, the reproductive phenotype must be highly advantageous for the host insect. The infection frequencies with *Spiroplasma* range from 0% to 40% in natural populations of the pea aphid [29], which falls within the range (1%–50%) observed in other insects [1]. For offsetting the high fitness reduction due to loss of male offspring, potential benefits associated with the male-killing phenotype have been investigated both theoretically and empirically, which include increment of available resources through cannibalism, avoidance of inbreeding depression, reduced local mate/resource competition, and others [40]. Previous studies on the pea aphid using molecular markers have shown that populations associated with perennial host plants tend to produce sexual forms and are generally less dispersive, while populations associated with annual host plants tend to produce few sexual forms and are more dispersive [28], [29]. Inbred crosses are thus more likely in the former type of populations than in the latter type of populations, especially after several generations of clonal reproduction on the same plant in the same field. It was certainly reported that *Spiroplasma* infections were higher in populations on perennial host plants (30–40%) than in populations on annual host plants (less than 10%) [29]. It is conceivable, although speculative, that *Spiroplasma* infection may reduce the risk of inbreeding depression through male-killing in such aphid populations. Indeed, breeding of clone mates or related individuals in *A. pisum* would certainly result in strong fitness reduction as documented in other aphid species [41], [42]. Whether the benefits of inbreeding avoidance are sufficient to offset the possible costs of the *Spiroplasma* infection during asexual phase of the life cycle requires further theoretical and experimental work. Evolutionary and population genetic models have suggested that under certain circumstances (e.g. imperfect maternal transmission, partial host resistance, etc.), a host-male killer equilibrium may be stably maintained without mutualistic interactions [2], [43], [44]. In this context, it is notable that some facultative symbionts (i.e. *Regiella insecticola*) of the pea aphid are not only maternally but also paternally transmitted [24].

### Facultative symbionts do not control reproductive mode variation in aphids

This study showed that the five facultative symbionts of the pea aphid did not influence the reproductive mode under the nine tested host genotypes. The cyclically parthenogenetic aphid genotypes (P33, P123, P136, YR2) kept their ability to produce sexual females and males irrespective of their symbiont composition (except for *Spiroplasma* infection). The permanently or predominantly parthenogenetic aphid genotypes (L100, 4TV, 10TV, T3-8V1) did not increase the sexual form production when infected with the facultative symbionts. This study extends the result of a previous work that *Rickettsia* infection does not affect the host reproductive mode under different pea aphid genotypes [25]. Hence, the partial or complete losses of the sexual function in some *A. pisum* genotypes, which have occurred in aphids very frequently, seem not due to their symbionts but rather due to structural or functional variations in their own genomes. This evolutionary perspective conforms earlier works using classical genetic approaches [45], [46] and more recent studies based on functional genomics and gene expression analyses [33], [47].

### Costs of symbiont infection during the sexual phase

In this study, we demonstrated that the host fitness components during the sexual phase are often negatively affected by infection with the facultative symbionts. Generally, symbiont transfection had a negative impact on the reproduction of the novel recipient host, as observed in a previous study that focused on only one symbiont (*Rickettsia*) and two aphid genotypes [25]. Reduced fecundities in infected lines could be, at least partly, explained by the recent and artificial nature of these novel symbioses [16], [48].

Among the facultative symbionts of the pea aphid, *Hamiltonella* induced the strongest fitness reduction regardless of the host genetic backgrounds into which it was transfected exhibiting shorter lifespan and reduced fecundity. Prevalence of this symbiont in natural populations of the pea aphid could reach up to 85%. Especially, in the races specialized on alfalfa, infection frequencies with *Hamiltonella* are 60–70% on average [29], [49]. Such a high prevalence despite strong infection costs could be explained by the well-known advantage of this symbiont in conferring protection against natural enemies [19], [50]. In addition, it could be that *Hamiltonella* infection is favoured in alfalfa specialized genotypes by enhancing host-plant utilization as demonstrated for some *Regiella*-infected lines feeding on clover [17], [51], but see [52] for contradictory results on other aphid lines. However, this hypothesis seems not supported by recent data on involvement of *Hamiltonella* in aphid plant adaptation [53]. Finally, the strong negative impact of *Hamiltonella* could be due to the injected strain: variation between strains of facultative symbionts has been showed for several infuenced traits [14], [50]

### Conclusions

Our data along with previous results on facultative symbionts of aphids shed a new light on the amazing diversity and complexity of the endosymbiotic interactions. Facultative symbionts affect their hosts through a wide range of mechanisms that influence distinct biological functions such as reproduction, development, nutrition, behaviour, pigmentation and resistance against biotic and abiotic stress. In addition, such microbial effects are modulated by strong symbiont-by-host genotype interactions, as illustrated here and in previous works [50], [54]. Horizontal transfers of such facultative symbionts among host species seem to be very common [7], [55], new facultative symbionts are regularly described [12], [13], [15], and, consequently, now we recognize the fact that a range of diverse facultative symbionts frequently coexist within the same host species and populations. Emerging questions are on the interactions between these microbes, on the interactions between obligate symbiont and facultative symbionts, and on the effects of these super-symbioses on the ecology and physiology of their hosts. As genome sequencing of these facultative symbionts progresses [56], [57] and more ecological, evolutionary and physiological studies on them are compiled, major advances will soon be made in understanding of interactions between multiple microbial partners in their host insects.

## Supporting Information

Table S1 **Means and standard errors (S.E.) of life-history and variables related to morph production measured in experiment 1 on isofemale lines of “sexual” and asexual genotypes of the pea aphid differing in composition of facultative symbionts.**
(DOC)

Table S2 **Means and standard errors (S.E.) of life-history and variables related to morph production measured in experiment 2 on isofemale lines of “sexual” and asexual genotypes of the pea aphid differing in composition of facultative symbionts.**
(DOC)

Table S3 **Generalised linear models showing the effects of infection status (composition in facultative symbionts) and the genotypes of** ***Acyrthosiphon pisum*** **on their reproductive life history traits measured in experiment 1.**
(DOC)
