# Supplementary material for: Facultative Symbiont Infections Affect Aphid Reproduction
Source: PLoS One. 2011 Jul 27;6(7):e21831. doi: 10.1371/journal.pone.0021831 (PMC3144876; doi:10.1371/journal.pone.0021831)
Supplement: Table S1 — Means and standard errors (S.E.) of life-history and variables related to morph production measured in experiment 1 on isofemale lines of “sexual” and asexual genotypes of the pea aphid differing in composition of facultative symbionts. (DOC) [file pone.0021831.s001.doc]

|  |  | Asexual genotypes | | | | | | | | | | "Sexual" genotypes | | | | | | | |
| --- | --- | --- | --- | --- | --- | --- | --- | --- | --- | --- | --- | --- | --- | --- | --- | --- | --- | --- | --- |
|  |  | 10TV | | 4TV | | L100 | | L1-22 | | T3-8V1 | | P123 | | P136 | | P33 | | YR2 | |
|  |  | Mean | S.E. | Mean | S.E. | Mean | S.E. | Mean | S.E. | Mean | S.E. | Mean | S.E. | Mean | S.E. | Mean | S.E. | Mean | S.E. |
| Age at first reproduction | *Buchnera* | 12.33 | 0.33 | 12.08 | 0.15 | 12.92 | 0.08 | 11.42 | 0.15 | 12.08 | 0.19 | 12.58 | 0.23 | 13.50 | 0.23 | 12.75 | 0.18 | 13.92 | 0.23 |
|  | *Hamiltonella* | 11.73 | 0.36 | 12.58 | 0.51 | 11.75 | 0.25 | 9.75 | 0.13 | 11.64 | 0.20 | 16.17 | 0.17 | 12.08 | 0.36 | 13.22 | 0.70 | 14.00 | 0.25 |
|  | *Regiella* | 12.17 | 0.41 | 12.18 | 0.23 | 9.00 | 0.17 | 15.83 | 0.11 | 12.00 | 0.23 | 13.00 | 0.28 | 12.33 | 0.19 | 12.08 | 0.43 | 13.50 | 0.15 |
|  | *Rickettsia* | 12.50 | 0.23 | 12.08 | 0.23 | 11.00 | 0.00 | 11.36 | 0.28 | 12.92 | 0.31 | 12.50 | 0.23 | 15.17 | 0.52 | 13.50 | 0.23 | 13.42 | 0.15 |
|  | *Serratia + Rickettsiella* | 12.08 | 0.29 | 12.00 | 0.21 | 15.33 | 0.62 | 16.17 | 0.39 | 11.75 | 0.18 | 11.92 | 0.26 | 13.25 | 0.18 | 12.58 | 0.34 | 14.33 | 0.51 |
|  | *Spiroplasma* | 11.58 | 0.34 | 11.75 | 0.22 | 13.25 | 0.25 | 10.64 | 0.15 | 11.58 | 0.23 | 12.92 | 0.08 | 13.00 | 0.30 | 12.42 | 0.19 | 13.27 | 0.27 |
| Reproductive lifespan | *Buchnera* | 16.75 | 1.16 | 19.33 | 0.57 | 26.92 | 1.02 | 20.25 | 1.24 | 18.42 | 0.62 | 19.83 | 1.43 | 20.33 | 0.90 | 20.08 | 0.73 | 23.17 | 1.22 |
|  | *Hamiltonella* | 4.18 | 0.66 | 5.75 | 0.60 | 6.13 | 1.13 | 14.58 | 1.67 | 7.45 | 0.25 | 4.83 | 0.17 | 3.33 | 0.56 | 4.78 | 1.60 | 9.08 | 0.91 |
|  | *Regiella* | 17.50 | 0.81 | 14.45 | 0.53 | 10.83 | 0.39 | 19.67 | 1.59 | 18.55 | 0.97 | 17.92 | 1.01 | 8.75 | 0.37 | 17.08 | 1.30 | 21.00 | 2.22 |
|  | *Rickettsia* | 18.25 | 1.88 | 19.33 | 0.67 | 28.00 | 1.56 | 20.18 | 1.48 | 22.50 | 1.38 | 18.00 | 0.84 | 15.00 | 1.21 | 16.83 | 0.61 | 20.67 | 0.89 |
|  | *Serratia + Rickettsiella* | 17.92 | 1.12 | 18.83 | 0.47 | 19.44 | 2.89 | 15.67 | 1.08 | 17.00 | 1.08 | 17.67 | 0.48 | 15.33 | 1.18 | 17.50 | 0.88 | 16.25 | 1.10 |
|  | *Spiroplasma* | 12.50 | 0.45 | 11.33 | 0.67 | 18.75 | 0.95 | 21.27 | 0.98 | 18.00 | 0.58 | 14.67 | 1.05 | 15.08 | 0.88 | 15.25 | 1.10 | 10.73 | 0.54 |
| Longevity | *Buchnera* | 31.50 | 1.29 | 35.00 | 0.59 | 44.00 | 0.64 | 36.75 | 1.84 | 32.33 | 1.02 | 37.58 | 1.25 | 43.75 | 1.38 | 37.83 | 0.73 | 43.33 | 1.55 |
|  | *Hamiltonella* | 17.09 | 0.67 | 20.08 | 0.51 | 18.88 | 0.99 | 28.58 | 1.31 | 20.27 | 0.33 | 22.67 | 0.67 | 16.92 | 0.47 | 18.89 | 2.03 | 27.42 | 0.65 |
|  | *Regiella* | 32.25 | 0.95 | 32.27 | 1.20 | 31.00 | 1.13 | 38.08 | 1.61 | 32.18 | 1.19 | 34.33 | 1.28 | 30.17 | 0.69 | 34.58 | 1.40 | 39.17 | 2.53 |
|  | *Rickettsia* | 34.08 | 2.18 | 34.83 | 0.86 | 43.17 | 1.25 | 32.91 | 1.76 | 37.83 | 1.86 | 35.25 | 0.91 | 40.92 | 1.38 | 35.08 | 0.51 | 40.67 | 1.07 |
|  | *Serratia + Rickettsiella* | 34.42 | 0.50 | 33.92 | 0.63 | 35.78 | 2.71 | 34.08 | 1.05 | 31.33 | 1.47 | 35.17 | 0.46 | 32.92 | 1.54 | 35.75 | 0.51 | 34.67 | 1.08 |
|  | *Spiroplasma* | 25.00 | 0.51 | 25.17 | 0.53 | 37.33 | 1.53 | 35.73 | 1.17 | 32.00 | 0.77 | 31.17 | 1.20 | 32.92 | 0.92 | 33.00 | 0.46 | 25.82 | 0.33 |
| Total fecundity | *Buchnera* | 78.08 | 5.51 | 106.17 | 1.84 | 65.08 | 5.13 | 69.50 | 5.65 | 123.33 | 4.53 | 61.75 | 2.18 | 57.42 | 4.30 | 51.83 | 3.52 | 77.67 | 6.25 |
|  | *Hamiltonella* | 18.00 | 3.76 | 31.00 | 4.36 | 22.25 | 6.29 | 53.67 | 5.95 | 29.64 | 2.39 | 13.50 | 1.88 | 10.08 | 1.68 | 12.00 | 2.32 | 30.08 | 3.15 |
|  | *Regiella* | 70.83 | 6.16 | 82.55 | 4.14 | 52.17 | 5.97 | 56.83 | 4.34 | 124.00 | 3.46 | 53.67 | 3.47 | 27.83 | 2.23 | 49.17 | 4.18 | 84.83 | 7.50 |
|  | *Rickettsia* | 74.92 | 7.77 | 100.92 | 4.13 | 61.50 | 5.44 | 46.09 | 5.85 | 107.00 | 3.66 | 59.08 | 3.31 | 47.17 | 5.06 | 42.83 | 2.93 | 73.75 | 4.74 |
|  | *Serratia + Rickettsiella* | 75.75 | 3.31 | 96.25 | 3.99 | 46.56 | 8.72 | 35.92 | 2.97 | 93.58 | 4.59 | 61.67 | 1.79 | 49.50 | 4.18 | 52.75 | 3.03 | 47.25 | 5.35 |
|  | *Spiroplasma* | 58.25 | 4.06 | 79.00 | 3.83 | 58.25 | 6.69 | 56.27 | 4.24 | 118.75 | 3.15 | 26.42 | 5.42 | 24.67 | 2.24 | 37.08 | 4.67 | 34.45 | 2.52 |
| Proportion of asexual females in the progeny | *Buchnera* | ND | ND | ND | ND | ND | ND | ND | ND | ND | ND | 0.08 | 0.04 | 0.23 | 0.05 | 0.21 | 0.06 | 0.43 | 0.06 |
|  | *Hamiltonella* | ND | ND | ND | ND | ND | ND | ND | ND | ND | ND | 0.18 | 0.16 | 0.00 | 0.00 | 0.00 | 0.00 | 0.02 | 0.01 |
|  | *Regiella* | ND | ND | ND | ND | ND | ND | ND | ND | ND | ND | 0.14 | 0.04 | 0.02 | 0.01 | 0.23 | 0.06 | 0.38 | 0.05 |
|  | *Rickettsia* | ND | ND | ND | ND | ND | ND | ND | ND | ND | ND | 0.08 | 0.03 | 0.31 | 0.04 | 0.17 | 0.04 | 0.33 | 0.04 |
|  | *Serratia + Rickettsiella* | ND | ND | ND | ND | ND | ND | ND | ND | ND | ND | 0.04 | 0.03 | 0.12 | 0.03 | 0.12 | 0.04 | 0.20 | 0.05 |
|  | *Spiroplasma* | ND | ND | ND | ND | ND | ND | ND | ND | ND | ND | 0.25 | 0.08 | 0.07 | 0.03 | 0.15 | 0.04 | 0.05 | 0.02 |
| Proportion of males in the progeny | *Buchnera* | ND | ND | ND | ND | 0.14 | 0.03 | ND | ND | ND | ND | 0.59 | 0.04 | 0.28 | 0.06 | 0.26 | 0.05 | 0.00 | 0.00 |
|  | *Hamiltonella* | ND | ND | ND | ND | 0.00 | 0.00 | ND | ND | ND | ND | 0.01 | 0.01 | 0.00 | 0.00 | 0.00 | 0.00 | 0.00 | 0.00 |
|  | *Regiella* | ND | ND | ND | ND | 0.00 | 0.00 | ND | ND | ND | ND | 0.41 | 0.06 | 0.33 | 0.04 | 0.22 | 0.04 | 0.00 | 0.00 |
|  | *Rickettsia* | ND | ND | ND | ND | 0.09 | 0.03 | ND | ND | ND | ND | 0.52 | 0.05 | 0.17 | 0.02 | 0.31 | 0.06 | 0.00 | 0.00 |
|  | *Serratia + Rickettsiella* | ND | ND | ND | ND | 0.03 | 0.01 | ND | ND | ND | ND | 0.62 | 0.03 | 0.22 | 0.04 | 0.42 | 0.06 | 0.01 | 0.01 |
|  | *Spiroplasma* | ND | ND | ND | ND | 0.00 | 0.00 | ND | ND | ND | ND | 0.00 | 0.00 | 0.00 | 0.00 | 0.00 | 0.00 | 0.00 | 0.00 |
