# Supplementary material for: Facultative Symbiont Infections Affect Aphid Reproduction
Source: PLoS One. 2011 Jul 27;6(7):e21831. doi: 10.1371/journal.pone.0021831 (PMC3144876; doi:10.1371/journal.pone.0021831)
Supplement: Table S2 — Means and standard errors (S.E.) of life-history and variables related to morph production measured in experiment 2 on isofemale lines of “sexual” and asexual genotypes of the pea aphid differing in composition of facultative symbionts. (DOC) [file pone.0021831.s002.doc]

|  |  | Pea aphid genotypes | | | | | | | |
| --- | --- | --- | --- | --- | --- | --- | --- | --- | --- |
|  |  | P123 | | P136 | | P33 | | YR2 | |
|  |  | Mean | S.E. | Mean | S.E. | Mean | S.E. | Mean | S.E. |
| Age at first reproduction | *Buchnera* | 12.08 | 0.15 | 12.17 | 0.24 | 11.92 | 0.23 | 11.42 | 0.47 |
|  | *Rickettsia* | 12.64 | 0.15 | 12.50 | 0.15 | 12.08 | 0.23 | 12.92 | 0.23 |
|  | *Spiroplasma* | 11.82 | 0.12 | 11.58 | 0.28 | 11.75 | 0.18 | 12.54 | 0.17 |
| Reproductive lifespan | *Buchnera* | 19.83 | 1.05 | 18.25 | 1.35 | 19.58 | 0.91 | 23.50 | 0.83 |
|  | *Rickettsia* | 17.73 | 0.99 | 17.83 | 0.63 | 14.92 | 0.83 | 22.08 | 0.93 |
|  | *Spiroplasma* | 15.18 | 1.35 | 13.04 | 0.59 | 12.58 | 1.25 | 8.04 | 0.47 |
| Longevity | *Buchnera* | 35.83 | 0.88 | 33.92 | 1.59 | 35.83 | 0.87 | 39.42 | 0.68 |
|  | *Rickettsia* | 34.09 | 1.40 | 35.33 | 1.02 | 31.33 | 0.36 | 39.17 | 1.33 |
|  | *Spiroplasma* | 30.55 | 0.64 | 27.71 | 0.37 | 31.17 | 0.88 | 22.50 | 0.36 |
| Total fecundity | *Buchnera* | 60.17 | 2.44 | 61.92 | 3.52 | 66.00 | 2.95 | 71.33 | 4.89 |
|  | *Rickettsia* | 57.09 | 3.63 | 56.42 | 2.77 | 40.17 | 2.56 | 85.67 | 3.20 |
|  | *Spiroplasma* | 21.18 | 1.98 | 44.21 | 3.16 | 32.33 | 4.53 | 28.42 | 2.77 |
| Proportion of asexual females in the progeny | *Buchnera* | 0.13 | 0.05 | 0.59 | 0.07 | 0.28 | 0.07 | 0.81 | 0.06 |
|  | *Rickettsia* | 0.26 | 0.05 | 0.62 | 0.08 | 0.40 | 0.08 | 0.82 | 0.03 |
|  | *Spiroplasma* | 0.96 | 0.04 | 0.85 | 0.04 | 0.44 | 0.11 | 0.93 | 0.04 |
| Proportion of males in the progeny | *Buchnera* | 0.62 | 0.02 | 0.27 | 0.06 | 0.50 | 0.04 | 0.13 | 0.05 |
|  | *Rickettsia* | 0.54 | 0.05 | 0.24 | 0.08 | 0.29 | 0.05 | 0.00 | 0.00 |
|  | *Spiroplasma* | 0.00 | 0.00 | 0.00 | 0.00 | 0.00 | 0.00 | 0.00 | 0.00 |
