# Supplementary material for: Facultative Symbiont Infections Affect Aphid Reproduction
Source: PLoS One. 2011 Jul 27;6(7):e21831. doi: 10.1371/journal.pone.0021831 (PMC3144876; doi:10.1371/journal.pone.0021831)
Supplement: Table S3 — Generalised linear models showing the effects of infection status (composition in facultative symbionts) and the genotypes of Acyrthosiphon pisum on their reproductive life history traits measured in experiment 1. (DOC) [file pone.0021831.s003.doc]

|  | Model elements | | | | | | | | | | |
| --- | --- | --- | --- | --- | --- | --- | --- | --- | --- | --- | --- |
|  | Aphid infection status(1) | | |  | Aphid genotype(2) | | |  | Interaction(1)×(2) | | |
| Dependent variables (distribution family) | *d.f.* | *F* | *P*-value |  | *d.f.* | *F* | *P*-value |  | *d.f.* | *F* | *P*-value |
|  |  |  |  |  |  |  |  |  |  |  |  |
| *(a) Asexual genotypes* |  |  |  |  |  |  |  |  |  |  |  |
| Age at first reproduction (Gamma) | 5 | 28.92 | <0.001 |  | 4 | 4.48 | <0.005 |  | 20 | 35.92 | <0.001 |
| Reproductive lifespan (Gamma) | 5 | 104.54 | <0.001 |  | 4 | 17.09 | <0.001 |  | 20 | 13.86 | <0.001 |
| Longevity (Gamma) | 5 | 125.41 | <0.001 |  | 4 | 33.45 | <0.001 |  | 20 | 10.72 | <0.001 |
| Total fecundity (Quasipoisson) | 5 | 76.77 | <0.001 |  | 4 | 83.11 | <0.001 |  | 20 | 7.55 | <0.001 |
|  |  |  |  |  |  |  |  |  |  |  |  |
|  |  |  |  |  |  |  |  |  |  |  |  |
| *(b) Sexual genotypes* |  |  |  |  |  |  |  |  |  |  |  |
| Age at first reproduction (Gamma) | 5 | 5.03 | <0.001 |  | 3 | 13.30 | <0.001 |  | 15 | 8.30 | <0.001 |
| Reproductive lifespan (Gamma) | 5 | 74.16 | <0.001 |  | 3 | 11.25 | <0.001 |  | 15 | 7.86 | <0.001 |
| Longevity (Gamma) | 5 | 131.69 | <0.001 |  | 3 | 4.87 | <0.005 |  | 15 | 12.14 | <0.001 |
| Total fecundity (Quasipoisson) | 5 | 76.31 | <0.001 |  | 3 | 34.27 | <0.001 |  | 15 | 5.97 | <0.001 |
| Proportion of asexual females in the progeny (Quasibinomial) | 5 | 17.66 | <0.001 |  | 3 | 30.18 | <0.001 |  | 15 | 4.54 | <0.001 |
| Proportion of males in the progeny (Quasibinomial) | 5 | 59.35 | < 0.001 |  | 3 | 264.60 | < 0.001 |  | 15 | 2.67 | < 0.001 |
|  |  |  |  |  |  |  |  |  |  |  |  |
